# Photochemically Induced Propulsion of a 4D Printed Liquid Crystal Elastomer Biomimetic Swimmer

**DOI:** 10.1002/advs.202308561

**Published:** 2024-04-08

**Authors:** Paolo Sartori, Rahul Singh Yadav, Jesús del Barrio, Antonio DeSimone, Carlos Sánchez‐Somolinos

**Affiliations:** ^1^ Instituto de Nanociencia y Materiales de Aragón (INMA), CSIC‐Universidad de Zaragoza Departamento de Física de la Materia Condensada Zaragoza 50009 Spain; ^2^ Instituto de Nanociencia y Materiales de Aragón (INMA), CSIC‐Universidad de Zaragoza Departamento de Química Orgánica Zaragoza 50009 Spain; ^3^ The BioRobotics Institute Scuola Superiore Sant'Anna Pisa 56127 Italy; ^4^ SISSA‐Scuola Internazionale Superiore di Studi Avanzati Trieste 34136 Italy; ^5^ Centro de Investigación Biomédica en Red de Bioingeniería, Biomateriales y Nanomedicina Instituto de Salud Carlos III Zaragoza 50018 Spain

**Keywords:** 4D printing, azobenzene, biomimetic swimmers, liquid crystal elastomers, photochemical actuation

## Abstract

Underwater organisms exhibit sophisticated propulsion mechanisms, enabling them to navigate fluid environments with exceptional dexterity. Recently, substantial efforts have focused on integrating these movements into soft robots using smart shape‐changing materials, particularly by using light for their propulsion and control. Nonetheless, challenges persist, including slow response times and the need of powerful light beams to actuate the robot. This last can result in unintended sample heating and potentially necessitate tracking specific actuation spots on the swimmer. To tackle these challenges, new azobenzene‐containing photopolymerizable inks are introduced, which can be processed by extrusion printing into liquid crystalline elastomer (LCE) elements of precise shape and morphology. These LCEs exhibit rapid and significant photomechanical response underwater, driven by moderate‐intensity ultraviolet (UV) and green light, being the actuation mechanism predominantly photochemical. Inspired by nature, a biomimetic four‐lapped ephyra‐like LCE swimmer is printed. The periodically illumination of the entire swimmer with moderate‐intensity UV and green light, induces synchronous lappet bending toward the light source and swimmer propulsion away from the light. The platform eliminates the need of localized laser beams and tracking systems to monitor the swimmer's motion through the fluid, making it a versatile tool for creating light‐fueled robotic LCE free‐swimmers.

## Graphical abstract

4D printing of azobenzene‐containing liquid crystal elastomers (LCEs) actuating at low temperatures close to room temperature is demonstrated. Elastomer irradiation with moderate‐intensity ultraviolet light leads to fast photodeformation underwater indicating predominant photochemical response. Periodic illumination of a 4D printed biomimetic four‐lapped ephyra‐like LCE swimmer, with moderate‐intensity UV and green light, covering the entire sample, propels the swimmer away the light source.

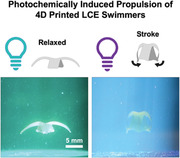

## 1 Introduction

Underwater organisms exhibit remarkable swimming abilities with high maneuverability. As a result, nature serves as an invaluable source of inspiration for soft robotics scientists who study, simulate, and reproduce these motions within their systems.^[^ ^1^, ^2^, ^3^, ^4^, ^5^ ^]^ Soft materials with shape‐changing abilities stand as essential components in advancing the capabilities of soft robotic swimmers. The integration of these materials has enabled researchers to replicate the swimming motions of natural organisms using a variety of external stimuli, including high‐power light,^[^ ^6^, ^7^, ^8^ ^]^ magnetic fields,^[^ ^9^ ^]^ or even cells and living tissues.^[^ ^10^, ^11^ ^]^ Among these shape‐shifting materials, hydrogels exhibit reversible deformation via water‐driven swelling and deswelling processes triggered by external stimuli.^[^ ^12^ ^]^ However, they generally exhibit relatively slow mass transfer and diffusion processes, a characteristic that may be problematic for swimming applications that necessitate rapid actuation.^[^ ^13^ ^]^ Another promising class of shape‐shifting materials are crosslinked liquid crystalline polymers (CLCPs). Either liquid crystalline networks (LCNs) or liquid crystal elastomers (LCEs), they typically show large anisotropic deformation due to the loss of molecular order within the material upon exposure to external triggers, causing the system to contract along the director, the direction of preferential molecular orientation.^[^ ^14^, ^15^, ^16^, ^17^ ^]^ Among the different stimuli to induce mechanical deformation in CLCPs, light stands out as particularly advantageous for swimmers due to its precise remote control, with exceptional spatial and temporal resolution. These systems harness light energy to initiate dynamic shape changes through two distinct modes of light‐induced mechanical deformation: photothermal and photochemical. In the photothermal mechanism, light is absorbed by nanoparticles or chromophores, either chemically bonded or dispersed in the liquid crystalline (LC) system, leading to the localized generation of heat. Consequently, the order of LC molecules is disrupted, resulting in macroscopic contraction along the director.^[^ ^18^, ^19^, ^20^, ^21^, ^22^, ^23^, ^24^, ^25^, ^26^ ^]^ When the LC system is in air, significant photoinduced heating and deformation can occur quite rapidly. However, if the LC system is in a fluid with higher thermal conductivity than air, the heat generated by the photothermal mechanism is efficiently taken away from the actuator.^[^ ^27^, ^28^, ^29^, ^30^ ^]^ As a result, the system needs a significantly higher amount of energy input to achieve the same temperature increase in the fluid when compared to experiments conducted in the air. Swimming capabilities have been successfully demonstrated using laser‐induced heating of absorbing liquid crystal gel (LCG) systems, however, a laser beam with an intensity of ≈2 W cm^−2^ needs to be targeted to the middle part of a monolithic rectangular strip made from this material.^[^ ^31^ ^]^ This localized heating induces fast bending of the LCG element, propelling it in water. However, sustained swimming requires a precise spatiotemporal tracking of the center of the LCG with the light of a relatively high‐power density that heats the element and the surrounding media.

Besides the photothermal effect, the photochemical mechanism, frequently introduced in CLCPs by incorporating azobenzene photo‐switches, offers promising possibilities.^[^ ^28^, ^32^, ^33^, ^34^, ^35^ ^]^ These chromophores can undergo light‐induced isomerization between the thermodynamically rod‐like promesogenic *trans* state and the bent‐shaped non‐mesogenic *cis* state that perturbs the LC order and induces a mechanical deformation of the material.^[^ ^33^ ^]^ This effect can even occur isothermally under moderate light intensities, especially when actuation is performed in fluids that efficiently dissipate heat from the sample. While these characteristics are intriguing, the slow photochemical response observed in the rigid crosslinked LCN matrices studied under these moderate light power densities, where response times range from tens of seconds to minutes, renders them unsuitable for applications in swimming systems. Consequently, underwater swimming based solely on the photochemically induced deformation of CLCPs has been rarely explored, with the only reported attempt involving a soft LC system moving forward inside a glass tube filled with water. The system is addressed by periodically alternating between ultraviolet (UV) light and white light of high intensity, in the order of 1 W cm^−2^. resulting in forward movement of the robot inside the tube that is slightly wider than the robot itself. The robot, with a total length of 26 mm, achieves a speed of 142 µm s^−1^.^[^ ^36^ ^]^

In this context, it becomes evident that there is a need to develop LC‐based systems that can rapidly respond to moderate light intensities to create functional free‐swimming systems. This will eliminate the need for high‐intensity light sources that are focused on specific spots on the swimmer, therefore requiring its tracking through the fluid. Preferably, the actuation should predominantly rely on photochemical mechanisms without involving heating of the sample and the environment. This aspect is particularly crucial, especially when we consider future applications of these systems in biology, where isothermal processes are highly desired. Furthermore, it would be advantageous to have access to materials and robust fabrication techniques that facilitate the creation of LC‐based structures with intricate shapes and director morphologies. These structures could then mimic the morphology and movements of aquatic organisms upon stimulation, thereby enabling the development of artificial LC‐based free‐swimmers.

In this paper, we aim to tackle the above‐mentioned challenges in the field of LC‐based light‐fueled swimmers. We achieve this by introducing novel azobenzene‐containing macromers with a liquid crystal to isotropic phase transition at low temperature. These macromers are processable through extrusion printing. Using this technique, we can align the deposited mesogens parallel to the nozzle movement direction, allowing us to digitally define the shape and director morphology of the deposited material. This material is then transformed into an LCE through photopolymerization. The resulting LCE exhibits a significant mechanical response underwater at temperatures slightly above room temperature (RT). Importantly for our purposes, the system also demonstrates rapid and substantial photo‐deformation when exposed to moderate‐intensity UV light (365 nm, 100 mW cm^−2^) underwater. Notably, the bending response persists even after the light is switched off, suggesting a primarily photochemical origin. The system can be efficiently reverted to the original state in few seconds by irradiating it with green light (505 nm, 40 mW cm^−2^). Drawing inspiration from nature, we have designed and fabricated a biomimetic LCE system using extrusion printing, closely resembling a four‐lapped ephyra. By exposing the system to periodic illumination, achieved through alternating UV and green light pulses of moderate intensity, we induce cycles of reversible bending of the lappets toward the light source underwater, followed by a slower unbending. The repeated bending motion results in the swimmer being propelled away from the UV light source with an average speed in the mm s^−1^ range. The LCE swimmer is illuminated entirely using conventional LEDs that cover a wide area, much wider than the swimmer, eliminating the need of localized laser beams and tracking systems to follow the swimmer's free motion through the fluid.

## 2 Results and Discussion

### 2.1 Biomimetic Inspiration

**Figure 1:**
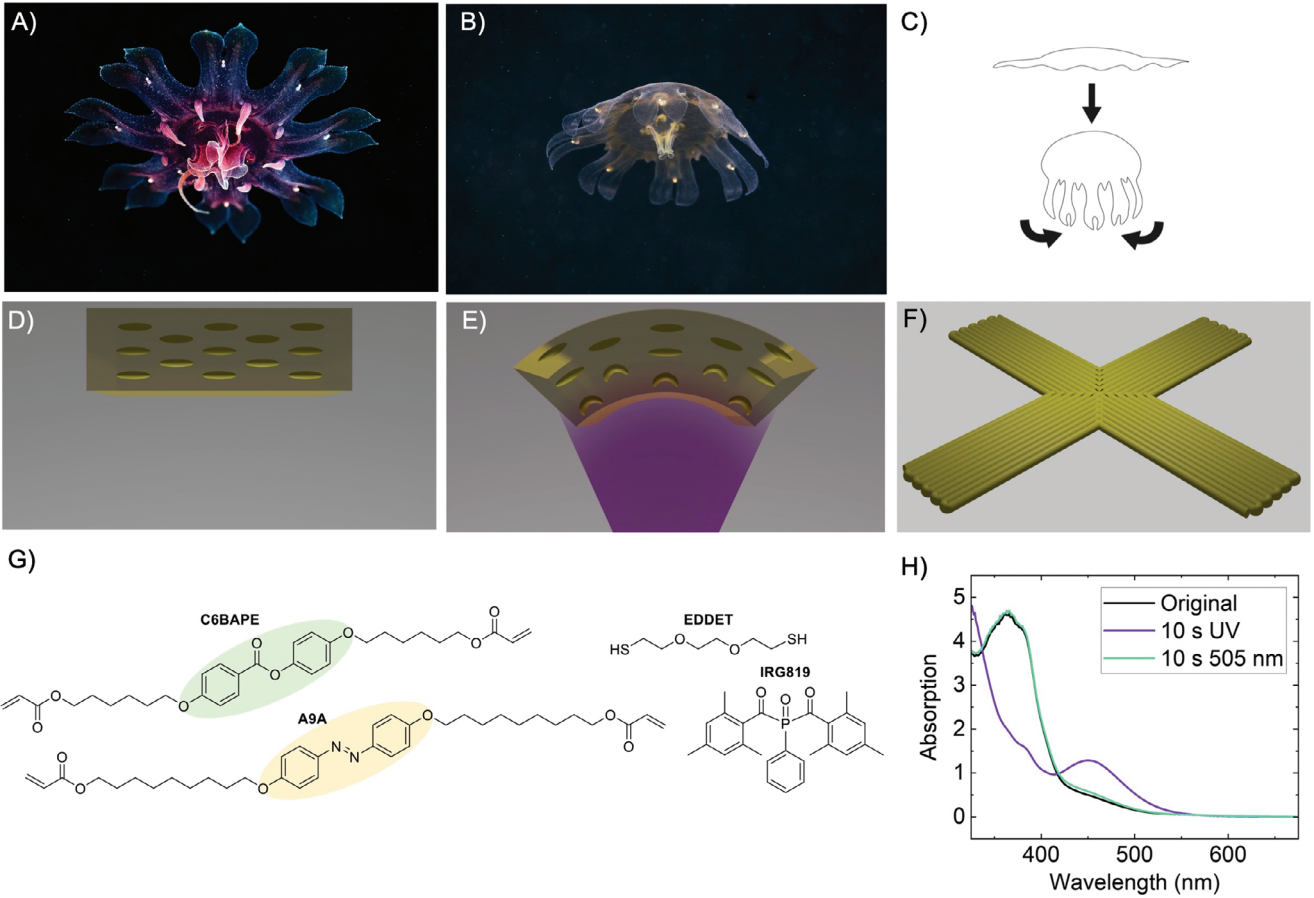
Biomimetic inspiration, swimmer design and LCE materials. A,B) Images of an ephyra, the organism used as the basis for the biomimetic design of our swimming system, in both A) a relaxed state and B) a contracted state (photos by Alexander Semenov, reproduced with permission of the author)^[^ ^45^, ^46^ ^]^ C) Schematic swimming process of an ephyra: the contraction of the bell, along with the bending of the external lappets, creates a water vortex beneath the organism, enabling it to propel itself. D,E) Bending principle of an azobenzene containing uniaxially oriented LCE strip. D) In the absence of previous irradiation, azobenzene are all in the trans state, the film being flat. E) When irradiated with UV light, the azobenzene molecules closer to the light source isomerize from the elongated trans to the bent cis form, while the ones further away will not be affected. This gradient in azobenzene isomers across the film thickness leads to film bending. F) Design of the printed biomimetic swimming system consisting of four LCE lappets with uniaxial alignment along the long direction that meet in the center in a square representation of a +1 disclination with radial director field. G) Chemical structure of the used chemicals in the synthesis of the printable ink. H) Absorption spectra of a 20 µm thick (15 mol% A9A LCE) printed sample. The black line corresponds to the original spectrum, before any irradiation, while the purple one shows the spectrum after 10 s of UV irradiation (365 nm, 100 mW cm^−2^). The green line represents the spectrum after being irradiated with green light (505 nm, 100 mW cm^−2^), immediately after the UV light exposure.

The biomimetic inspiration to implement our swimmers came from the ephyra (**Figure** **1**a), an early development stage of a particular jellyfish class called scyphozoans.^[^ ^37^ ^]^ Most of young ephyrae have a discontinuous bell morphology having a central disk with 8 radially emerging lappets, separated by gaps.^[^ ^38^ ^]^ Such organisms evolve growing the central disk and widening the lappets until they fuse leading to a continuous umbrella‐like form of the adult medusae.^[^ ^39^ ^]^ The swimming of both developmental stages is based on a paddling‐based propulsion. In the case of ephyra, the swimming motion is accomplished through a rapid bell contraction, which constitutes the power stroke (depicted in Figure 1B), followed by a slower relaxation of the bell, known as the recovery stroke.^[^ ^40^ ^]^ During the bell contraction, the lappets undergo a significant bending away from their initial nearly flat orientation within the primary plane of the ephyra body. This pulsation can form a vortex of water underneath the ephyra (Figure 1C), and the organism is propelled by vortex shedding.

Here we focus, for our soft‐bodied swimmer, on an ephyra‐like structure with LCE bending lappets. This choice is grounded in the demonstrated ability of LC crosslinked systems, our selected materials, to effectively perform bending motions under stimuli.^[^ ^41^ ^]^ We have chosen light as stimulus to implement this mechanical function in our LCEs due to the high degree of spatial‐temporal control of the excitation, with no contact.^[^ ^8^, ^19^, ^21^, ^25^, ^32^, ^33^, ^34^, ^42^, ^43^ ^]^ To generate bending in the LCE material we exploit azobenzene molecules having long‐lived, bent‐shaped *cis* isomers able to disrupt the molecular order and generate stresses within the material. As a simple realization of the bending movement, we have opted for a rectangular strip of LCE containing azobenzene with uniaxial orientation of the director along the long axis of the element.^[^ ^44^ ^]^ In the absence of any previous irradiation, azobenzene molecules are all in the elongated, mesogenic *trans* state, being the film flat (Figure 1D); under UV light irradiation, these azobenzene‐containing LC crosslinked systems tend to bend toward the incident light. This effect is attributed to the excitation light gradient within the strip. Irradiation of the element generates bent‐shaped, non‐mesogenic *cis* isomers. However, the population of these isomers is expected to be greater on the irradiated side of the film than in deeper regions. This difference arises due to the high extinction coefficient of azobenzene molecules, which attenuate the UV light as it progresses through the strip thickness. The irradiated side, therefore, with a larger content of isomers in the *cis* state, experiences a larger photoinduced decrease of order, and tends to contract more than the non‐irradiated side of the sample (Figure 1E). These differences lead to stresses inside the sample that finally result in its bending toward the UV light source. The non‐mesogenic *cis* isomers can relax into the mesogenic *trans* isomer form either thermally or photochemically, by irradiating them with blue–green light. As a result, the stresses within the film disappear and the element recovers the initial flat shape. In addition to the described photochemical effects that can lead to mechanical deformation, the sample can also experience localized temperature increases due to light absorption. This leads to photothermally induced local changes in the LC order and subsequent mechanical deformation. However, we anticipate that these thermal effects are diminished when the sample is submerged in water, which efficiently dissipates heat due to its high thermal conductivity.

Having in mind these considerations and to demonstrate the swimming mechanism, we propose a simplified design of an ephyra‐like LCE structure having four lappets with uniaxial alignment along the long direction. We have reduced the number of lappets from the eight, which the real ephyra has, to four in order to facilitate the design (Figure 1F). Supporting our simplified design, it has been observed in nature that young jellyfish, even when their arms are amputated, are capable of reorganizing their remaining arms and rebuild their muscular network to restore their body symmetry, all without regenerating the lost arms. Despite having a reduced number of arms, these young jellyfish are able to maintain their swimming capability.^[^ ^38^ ^]^ The central region, where the four lappets intersect, presents a director pattern that is a square representation of a +1 disclination with a radial director field. Homogeneous UV irradiation of such an element from one side is expected to lead to concurrent bending of the four lappets toward the light source that we expect can produce displacement of the surrounding fluid and underwater locomotion.

### 2.2 Ink Synthesis, Characterization, and Printing of LCEs

Direct ink writing of photocurable LC macromers has proven to be a powerful strategy for generating complex patterns of responsive LCE.^[^ ^47^, ^48^, ^49^, ^50^, ^51^ ^]^ In the search for a suitable material for our swimmer, our objective was to develop an extrusion printable ink that, upon curing, forms a soft LCE capable of rapid underwater actuation when exposed to moderate‐intensity light. Our printable ink is composed of an acrylate‐ended main‐chain LC macromer and a photo‐initiator (IRG819). To introduce photochemical responsiveness, diacrylate A9A, which is a derivative of 4,4′‐dihydroxyazobenzene, has been selected (see Figure 1G).^[^ ^52^ ^]^ Azobenzene units, when incorporated into the main chain of LCEs, have demonstrated to be effective transducers for light induced mechanical actuation.^[^ ^28^, ^29^, ^53^ ^]^

To achieve effective and fast actuation of the printed elements, we have adopted a strategy of reducing the liquid crystal to isotropic transition (T_LC‐I_) of the LCE, bringing it closer to RT, the temperature at which we have performed the photoinduced swimming experiments. According to Warner, Finkelmann, Terentjev and other authors, UV irradiation of a liquid crystalline rubber containing azobenzene chromophores induces a change in the concentration of *cis* species, equivalent to an effective shift of the critical temperature of the underlying liquid crystal to isotropic phase transition.^[^ ^54^, ^55^, ^56^ ^]^ Performing photoinduced deformation experiments at RT, particularly in the vicinity of the critical temperature of the all‐*trans* material, is strategic. Under these conditions, UV irradiation induces an increase in *cis* isomer concentration, and this should take the system through the transition into the isotropic state, allowing for large deformations.

To generate such material we have incorporated a LC diacrylate (C6BAPE) into the main chain, with a mesogenic core comprising only two phenyl groups, an approach proven effective by Bauman et al.^[^ ^57^ ^]^ As stated by these authors, the weaker π–π interactions between mesogens in the polymer network, in comparison to the most commonly used mesogens with three phenyl groups (such as the well‐known RM82 or RM257), are responsible for the reduction in actuation temperature and the resulting increase in actuation rate at relatively low temperatures. Bearing this in mind, we have synthetized our macromers following a simple thiol‐acrylate Michael addition method of 2,2′‐(ethylenedioxy)diethanethiol (EDDET) to two different diacrylates, C6BAPE and A9A. We have employed a 1:1.15 molar ratio between EDDET and diacrylates constant in all the prepared precursors. In all the prepared macromers the excess of acrylate groups with respect to the thiol ensures that synthesized polymeric chains retain the acrylate groups at their ends as checked by ^1^H nuclear magnetic resonance (NMR) (see Figure S1, Supporting Information). Such groups will be reacted, after ink printing and via photopolymerization, to yield the final LCE. We explored the impact of azobenzene content on our materials by synthesizing three distinct macromers with varying percentages of azobenzene molecules, resulting in the preparation of three corresponding inks. To synthesize the macromer, we adjusted the relative proportion of azobenzene diacrylate molecules, namely 5, 10, and 15 mol% of A9A in relation to the total diacrylate content in the polymerization mixture. For the sake of simplicity throughout the manuscript, we will denote these corresponding inks and the resultant LCEs by referencing the molar percentage of A9A with respect to the diacrylate content. Details on the synthesis of the macromers and ink preparation can be found in the experimental section.^[^ ^58^ ^]^^13^C NMR as well as FTIR spectroscopic characterization has also been conducted on the inks (see Figures S2 and S3, Supporting Information, respectively).

All the inks display thermotropic behavior and a T_LC‐I_ at ca. 35 to 45 °C upon heating, according to our differential scanning calorimetry (DSC) measurements and polarization optical microscope (POM) observations (Figures S4 and S5, respectively). The glass transition temperature (T_g_) of all the inks is well below RT, at ca. −34 to −32 °C.

The LCE elements were prepared using an extrusion‐based 3D printer developed in our laboratory (see Experimental Section). The ink, contained in a temperature‐controlled printhead preset to 75 °C, was extruded and deposited on glass substrates coated with a thin layer of polyvinylalcohol (PVA), and kept at RT. Once the sample is printed, a curing step was performed to turn the macromer into an LCE. This was done by photoinduced polymerization with blue light of 430 nm. Gel fraction tests of the so cured samples were carried out in THF (see Experimental Section) leading to values higher than 95%. Besides, FTIR characterization of the ink and the cured LCE sample (see Figure S3, Supporting Information) shows that the band at ca. 1635–1640 cm^−1^, characteristic of the ─C═C─ bond of the acrylate groups, that is present in the spectrum of the ink, is absent in that of the LCE. This difference between the spectra of the uncured and cured sample, as well as the substantial value of the gel fraction, indicates the effective polymerization of the acrylate groups and formation of the LCE. After curing of one layer, the printing process could be repeated layer by layer, as detailed in the experimental section, leading to multilayer thicker elements.

Rectangular strips of ink were first deposited by printing closely packed lines parallel to the long axis of the rectangle. The lines fuse together leading to a continuous film that shows preferential alignment along the printed lines, as confirmed by observing the samples using a POM. Figure S6 (Supporting Information) presents POM images of the cured sample taken with the printing direction at 0° and at 45° with respect to the first polarizer transmission direction. The sample appears darker when the direction of the light polarization of the microscope aligns in parallel with the orientation of the printed fibers. Conversely, it becomes brighter as the angle changes, reaching its maximum brightness at 45°. These POM images, suggest a director alignment along the printing direction. Shear and elongational stresses during the printing process are the responsible for the alignment of the polymeric chains and therefore the mesogenic moieties along the needle movement direction.^[^ ^47^, ^48^, ^49^ ^]^

Figure 1H shows the UV–vis absorption spectrum of a LCE film (20 µm thick) with 15 mol% of azobenzene and uniaxially oriented director along the long axis. The azobenzene moiety in our inks shows two absorption peaks for the thermodynamically stable *trans* isomer (black line spectrum). The intense band in the UV region of the spectrum corresponds to the π∓π* transition, while the weaker one in the blue region is attributed to the n‐π* transition.^[^ ^52^ ^]^ Before any irradiation, with all the azobenzene in this *trans* form, the film presents a yellow color. After 10 s of UV irradiation with a moderate intensity of UV light (100 W cm ^−2^), the sample has turned orange in color and the shape of the spectrum drastically changes (purple line spectrum). The band in the UV region at 350–400 nm, almost has disappeared with respect the original, non‐irradiated film, while the band at ≈450 nm in the blue is more intense. It is well known that for such chromophores, these changes are associated with the photogeneration of *cis* isomers, with long half‐life times, typically of >1 h in LC matrices.^[^ ^19^ ^]^ The band associated to the π‐π* transition of the *cis* isomer is less intense than of the *trans* one, while the band corresponding to the n‐π* transition of the *cis* isomer is more intense than the one corresponding to the *trans* isomer, although remains essentially at the same position. It is also important to note that the substantial presence of *cis* isomers, after UV irradiation, strongly influences the mechanical properties of the material. A noticeable decrease in the elastic modulus of the uniaxial actuators has been observed, transitioning from 3.5 MPa for the uniaxial stripe containing 15 mol% of azobenzene before UV irradiation—where all the azobenzene molecules are in the *trans* form—to 1.1 MPa after UV irradiation. Irradiation of the system with green light of 505 nm after the UV exposure step has demonstrated to efficiently induce the photoisomerization of the *cis* isomers back to the *trans* state. The green line in figure 1H corresponds to the spectrum of the film after 10 s of moderate‐intensity green light (100 mW cm^−2^). Overall, these results indicate that UV and green light are appropriate wavelengths to rapidly and efficiently photoinduce the transformation between *trans* and *cis* isomers in our material. This swift photoinduced switching between isomers is expected to provide the fast photomechanical response too, which is required for swimming.

### 2.3 Thermo‐ and Photo‐Mechanical Response of LCE Elements in Water

**Figure 2:**
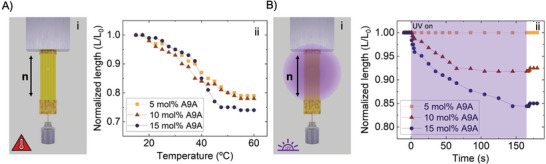
Underwater thermomechanical and photomechanical actuation under load. A 1 g weight is attached to one end of the uniaxially oriented LCE strips (60 ± 5 µm thick) with the director n along their long axis, while they are submerged in water. A‐i) Thermomechanical response. The load is lifted upon heating. ii) Normalized length (L/L_0_) of an LCE strip upon heating from 15 to 60 °C, for the three different printed materials (5, 10, and 15 mol% A9A). B‐i) Photomechanical response. The load is lifted upon irradiation with UV light. ii) Normalized length (L/L_0_) of an LCE strip during UV irradiation with 100 mWcm^−2^ for 160 s, for the three different printed materials (5, 10, and 15 mol% A9A). A partial recovery of the length is observed when UV illumination ceases in some of the samples.

Before conducting photomechanical studies on our printed actuators, the thermomechanical response of the uniaxially aligned LCE systems was assessed as a reference study. This evaluation was carried out in water, considering the final application of our systems as swimmers. Underwater thermoactuation tests were performed for LCE samples prepared with different azobenzene content (by using inks with 5, 10, and 15 mol% A9A). Uniaxially aligned LCE samples, 60 µm thick and consisting of three layers, were prepared following the procedure described in the experimental section. This sample thickness was selected based on its optimum performance in bending experiments (vide infra). The samples, after photopolymerization, are released from the substrate by dissolving the PVA intermediate sacrificial layer in water. After attaching an adhesive tape to one end of the sample, this is fixed to the sample holder from one end and a steel weight of 1 g was hung from the other one. The sample is immersed in a fluid contained in a temperature‐controlled chamber with optical access to visualize the sample. Samples were heated from 15 to 60 °C while immersed in the fluid and the length of the sample was monitored at different temperatures. **Figure** **2**A illustrates that all the samples exhibited significant contraction along the director when heated at temperatures above 17.5 °C. This contraction is attributed to a decrease in the mesogenic order, as previously described in the literature for uniaxially aligned LCE samples.^[^ ^14^ ^]^ In the temperature range of ≈30 to 35 °C, an increase in the rate of contraction per degree of temperature is observed, reaching, at 50 °C, a maximum contraction of >20% of their initial length. Notably, the sample with highest amount of azobenzene achieved the largest contraction, along with a higher rate of contraction per degree of temperature between 35 and 50 °C. Overall, these results underscore the low actuation temperature of the developed materials, consistent with previous research results concerning C6BAPE‐containing LCEs.^[^ ^57^ ^]^

Once the thermomechanical response of our LCE strips was assessed, and using the same setup, a contraction test under UV irradiation was performed, again for strips prepared with the 3 different inks (5,10, and 15 mol% A9A). For the whole experiment the samples are submerged in water kept at constant temperature (Figure 2B). As for the thermomechanical experiments, a weight of 1 g was attached to the lower end of the sample for the photomechanical experiments. In this case, this weight is added to prevent bending deformation during UV light irradiation, ensuring accurate measurement of the dimensions and shape changes of the LCE actuators. While the LCE strip prepared with the 5 mol% A9A ink did not display any measurable contraction during the entire irradiation period, both the 10 mol% and the 15 mol% A9A inks resulted in actuators that exhibited significant contractions: 8 and 16% of their original length, respectively, measured after 160 s of continuous UV irradiation (100 mW cm^−2^). Regarding the dynamics, a fast initial contraction is observed for both samples. After the first 5 s, the 15 mol% A9A sample has already contracted by 5% of its original length, while a 2% contraction is measured for the 10 mol% A9A at the same time. Upon irradiation, the contraction monotonically increases and reaches its maximum contraction after 145 s for the 15 mol% A9A sample, while the 10 mol% A9A sample achieves complete contraction in just 105 s. After switching off the UV illumination, a slight partial recovery of the length of the LCE strips is observed within the next few seconds. Both the 10 mol% A9A and 15 mol% A9A samples exhibit a slight decrease of the contraction of ≈1 to 2%, while still maintaining a significant remnant contraction value. Subsequent irradiation of the sample with green light (100 mW cm^−2^) led to a complete recovery of the initial length of the sample.

Photomechanical response in azobenzene LCEs is typically explained on the basis of photochemical and photothermal effects as previously described. On one side, UV irradiation of azobenzene molecules leads to efficient conversion of *trans* into *cis* isomers that are stable over time (see Figure 1H). The large conformational change of the azobenzene molecule upon *trans* to *cis* isomerization leads to an orientational order decrease of the LCE and thus a local contraction along the director direction and an expansion perpendicular to it. This could account for the phenomenology observed during the UV irradiation step for 10 and 15 mol% A9A LCEs while the photoinduced mechanical stresses are not sufficient to raise the 1 g load for the 5 mol% A9A sample. The slight relaxation of the contraction observed when stopping UV irradiation for 10 and 15 mol% A9A samples, can be attributed to the thermal relaxation of the sample that quickly reaches the temperature of the fluid again.^[^ ^28^, ^29^ ^]^ With this small difference in contraction, ascribed to the photoinduced heating of the sample during UV irradiation, the rest of the sample contraction is then related to the photochemical contribution due to presence of long‐lifetime *cis* isomers. These results suggest that, in our experimental conditions, for the samples with 10 and 15 mol% of azobenzene, the photochemical contribution dominates the photoinduced deformation, over the photothermal effect. The samples with higher azobenzene content are expected to generate upon UV irradiation a higher density of *cis* isomers thus leading to a higher contraction as experimentally seen with the larger deformation achieved for samples with 15 mol% A9A. The longer time required for the sample with higher azobenzene content could be attributed to greater absorption of the 15 mol% A9A, resulting in lower intensity reaching the deeper layers of the strip within a given time. Consequently, a higher total light dose is needed to excite the entire film and achieve maximum contraction. Subsequent illumination with green light leads to an initial recovery of the original length due to the efficient transformation of *cis* isomers to thermodynamically stable *trans* isomers, as demonstrated in our previous spectroscopic studies (see Figure 1H).

To achieve our goal of demonstrating free‐swimming capability of our printed systems, it is crucial to investigate the bending performance of our LCE strips underwater and without any external additional load. This bending mode is essential as it emulates the lappet paddling‐based propulsion of ephyrae. From this moment on, the experiments presented were performed with samples prepared with the ink having higher azobenzene content (15 mol% A9A), as they showed sufficiently fast and larger photochemical response in water.

**Figure 3:**
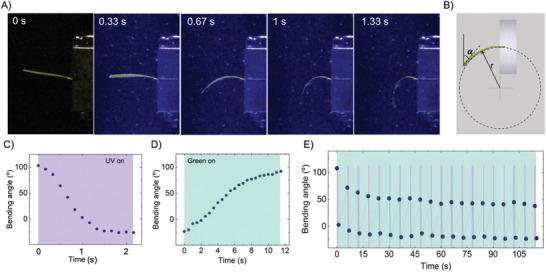
Underwater photoinduced bending. A three‐layer strip (60 µm thick) of 15 mol% A9A LCE with uniaxially oriented director along the long side of the actuator is held from one extreme to the sample holder. UV light reaches the sample from the bottom. A) Video frames captured at different irradiation times show the rapid bending of the sample. B) Angle between the tip of the actuator (the 10% length segment of the actuator at its free end) with the vertical direction, and schematization of the curvature radius (r) calculated by Timoshenko's formula for curvature in bilayers. C,D) Angle of the tip as a function of time during irradiation with C) UV 365 nm light; and D) subsequent green 505 nm light. E) Cyclic photoinduced bending and relaxation (20 cycles). In this experiment, the green light stays continuously switched on, while the UV light cycles on for one second followed by five seconds off.

The first test consisted in subjecting a strip with a uniaxially oriented director along the actuator's long side, held from one extreme to the sample holder, to continuous UV irradiation while submerged underwater. As shown in **Figure** **3**A, the three‐layer sample rapidly bends upon irradiation toward the UV light (100 mW cm^−2^) that reaches the sample from the bottom. This behavior can be attributed to the gradient of *cis* isomers across the film thickness, as described above. The angle formed by the tip of the actuator with the vertical direction, as illustrated in Figure 3B, is presented as a function of time in Figure 3C for the duration of the UV irradiation period. In just 1.5 s, the sample reaches its maximum equilibrium bending. Remarkably, within the first second, the sample becomes aligned parallel to the direction of light propagation. As anticipated above, this bending speed resulted to be optimal for this three‐layer sample. While the samples with one and two layers were bending to some extent under their own weight in water being therefore not valid for paddling, the sample with four layers resulted in a slower response (3.4 s to reach its maximum equilibrium bending) as shown in Figure S7 (Supporting Information). As expected, bending speed is also dependent on the UV light intensity, with higher intensities leading to higher bending speeds, although a similar dose is needed to reach certain bending angle regardless the intensity, this being consistent with a bending predominantly produced through a photochemical effect (See Figure S8, Supporting Information). After switching off the UV light, the sample maintains essentially the same deformation that was reached at the end of the UV irradiation (as seen in Movie S1, Supporting Information). This shape persistence further supports that the actuation is predominantly photochemical under our experimental conditions. The sample was then irradiated with green light (40 mW cm^−2^) collinear to the UV one. Figure 3D shows the angle of the tip actuator under these irradiation conditions. The sample recovers its initial configuration under green light irradiation, although this relaxation process is slower than the UV‐induced bending. However, the speed of this shape morphing could be easily tailored by adjusting the intensity of the light beams used. We anticipate that these differences in bending speed will be crucial for the subsequent stages of our study, particularly in the development of our swimmers. It is essential for the power stroke to be faster than the recovery stroke to achieve effective swimming capability.

To obtain a qualitative confirmation of the hypothesis that the bending deformations in Figure 3A are of photochemical origin, we use the Timoshenko's formula for curvature (1/*r*) in bilayers:^[^ ^59^ ^]^

(1) $$\;\frac{1}{r}=\;\frac{6\varepsilon_{p}{\left(1+m\right)}^{2}}{t\left(3{\left(1+m\right)}^{2}+\left(1+m\right)\left(m^{2}+\frac{1}{m}\right)\right)}$$

where *r* is the radius of curvature, *m*  =  *d*/*t* is the ratio between light penetration length scale *d* and film thickness *t*, and ε_*p*_ is the spontaneous strain defined as *L*/*L*_0_ − 1. In fact, using *d* = 10 µm and variable values for ε_*p*_ in the order of 0.01, the order of magnitude of relative length changes experienced by the sample of Figure 2B in the first 1s of illumination, we obtain Table S1 (see Supporting Information) which reproduces well the curvatures observed in Figure 3A (see also Figure S9, Supporting Information) and confirms the soundness of our hypothesis.

The second part of the test involved cyclically irradiating the sample (20 cycles) with UV light, while maintaining a constant level of intensity for the green light throughout the entire experiment. This was done for the sake of experimental simplicity. Within each cycle, the UV light was activated for 1 s and then turned off for 5 s. These illumination conditions demonstrate the repeatability of the actuation, as shown in Figure 3E. After the initial four stabilization cycles the magnitude of the bending at the beginning of the cycle (UV light switches on) and at end of the UV exposure (UV light switches off) is nearly constant for the remaining measured cycles (16) (see Movie S2, Supporting Information). After the set of cycles, the material is capable of recovering the initial configuration with a longer irradiation period under green light (505 nm).

### 2.4 Photochemical Induced Swimming

**Figure 4:**
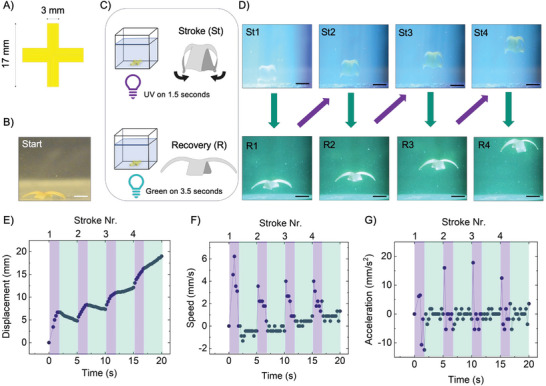
Photoinduced swimming of the biomimetic ephyra‐like LCE swimmer. Experimental setup and swimming mode characterization. A) Schematic view of the cross‐shape sample resembling a four‐lapped ephyra. Dimensions and director orientation are indicated. B) The printed ephyra‐like LCE swimmer is situated on the bottom of a fish tank in its original shape before any irradiation (scale bar: 5 mm). C) The setup consists of the cubic fish tank (25 cm side) filled with the fluid and two light sources (UV at 365 nm and Green at 505 nm) that collinearly illuminate the sample from the bottom. The UV illumination, taking 1.5 s, leads to a bending of the lappets, the stroke (St) phase, while the green light irradiation, taking 3.5 s, leads to the relaxation of the sample to the original shape, the recovery (R) phase. D) Four cycles of illumination of the LCE swimmer, each cycle consisting of UV exposure followed by the green illumination. Pictures are the last video frame of each irradiation step for UV (St1 to St4) and green (R1 to R4) light (scale bar: 5 mm). E–G) Swimming mode characterization: E) vertical position, F) speed, and G) acceleration as a function of time. The violet and green backgrounds visually show the UV and green irradiation periods of the system respectively.

Once the bending mode is characterized underwater, our final proof of concept was to demonstrate the free photoinduced swimming of a biomimetic ephyra‐like LCE system, as conceptually described in Figure 1C. The swimmer features four lappets arranged in a cross‐shaped geometry, with an arm span (from lappet tip to lappet tip) of 17 mm, as depicted in **Figure** **4**A. As previously described, the lappets have a uniaxial director orientation along their long axis. Consequently, UV irradiation of one side of the swimmer is expected to result in synchronous bending of the lappets.

Prior to conducting the light‐induced propulsion experiments of the swimmer, the density of the water was increased with the addition of sugar. This adjustment aimed to increase the buoyant force acting on the artificial swimmer, that is the upward force exerted by the fluid that equals the weight of the displaced fluid. A preliminary investigation involved testing various concentrations of sugar to determine the optimal level. The goal was to find a concentration at which the sample, when delicately suspended in the fluid, gradually sank to the tank's bottom under the influence of gravity. This provided a small downward force on the swimmer, slightly surpassing buoyancy. Maintaining a net force close to zero on the swimmer proved essential for facilitating photoinduced swimming upon irradiation. This was achieved despite resistance forces stemming from the viscosity of the water‐sugar solution at the identified optimum concentration, measured to be 3.8 cP.

The sample initially rests lying in a nearly flat morphology on the bottom of the tank (Figure 4B). Trying to implement swimming in this system, we explored the cyclic realization of fast lappet bending, constituting the power stroke, followed by a gradual relaxation of the bending back to the original state, known as the recovery stroke (Figure 4C). For the power stroke, the entire swimmer was irradiated under homogeneous 100 mW cm^−2^ UV light for 1.5 s, while for the recovery stroke, 40 mW cm^−2^ green light was shined on the sample for 3.5 s. Movie S3 (Supporting Information) shows the swimmer's movement in the fluid over several irradiation cycles. Figure 4D shows the last video frame of each irradiation step for the first four cycles. Figure 4E–G displays the instantaneous vertical position, speed, and acceleration of the swimmer, respectively. These values were calculated by analyzing the frames of Movie S3 (Supporting Information) of the experiment, as detailed in the Supporting Information, with the top apex serving as the reference point.

When irradiated with UV light for the first time, the sample is quick to move away from the bottom surface of the glass container, achieving a maximum displacement of 6 mm by the end of this initial stroke. At the beginning of this power stroke, the system quickly accelerates upward, resulting in increased speed. Notably, a considerable displacement, speed increase and acceleration are achieved within the first 250 ms of UV irradiation, once again highlighting the fast reaction response of the material when submerged underwater. The swimmer´s acceleration is attributed to hydrodynamic forces resulting from bending deformation, which in turn generates upward thrust. However, under UV light irradiation, the swimmer acceleration diminishes and even becomes negative. This is due to the absence of additional bending, which leads to a lack of propulsion. As a result, the effect of gravity becomes noticeable, causing the swimmer to be pulled down.

In the green light irradiation step, that is, the recovery stroke, the system gradually returns nearly its original shape, preparing for the subsequent UV stroke of the second cycle. Throughout the entire first cycle, the system reached a total height of ≈4 mm.

During the second cycle, the system followed similar dynamics. However, the overall increase in height achieved upon UV exposure was lower than in the first cycle, which can be attributed to the greater distance from the tank base. Similarly, in this case, the speed became negative during the recovery phase; however, the decrease in height was smaller compared to that of the first cycle. Once again, a net increase in height was attained throughout the entire cycle.

In contrast to the initial two illumination cycles, where a slight decrease in height was observed during the relaxation phase, the third and subsequent cycles showcase distinct behavior. During these later cycles, the swimmer has gained enough momentum to ascend even while in the relaxation phase. Overall, a displacement of 19 mm was achieved in the first 4 strokes, taking 20 s in total. The average speed in this period of time was 0.95 mm s^−1^, with the highest speed reaching a few mm s^−1^ at the beginning of each contraction phase in every cycle. After the completion of the four cycles, once both lights are switched off, the sample returned back to the bottom of the tank. This happened following a short period of time during which the sample continued to rise due to inertia, even without any stimuli.

## 3 Conclusion

In this work, azobenzene containing macromers with low liquid crystal to isotropic transition temperature have been developed. Photopolymerizable inks using these LC macromers can be deposited via extrusion printing leading to alignment of the mesogens parallel to the direction of movement of the needle. The digitally imprinted molecular morphology can be efficiently fixed into an LCE by photopolymerization while keeping well‐defined director morphology. Multiple layers can be added to the material while maintaining precise alignment in each printed layer, resulting in thicker LCE actuators. The resulting LCEs are capable of significant contraction along the director due to the heat induced loss of order of the mesogens. This contraction takes place when heated at temperatures slightly above RT, when underwater, with full contraction observed below 50 °C.

Under moderate‐intensity UV light emitted by an LED, LCEs containing 15 mol% azobenzene, subjected to a small load, exhibited significant 16% contraction from their initial length when underwater. This contraction remains largely even intact after UV light is switched off, highlighting the dominant photochemical effect in the deformation. Remarkably, the photothermal effect is substantially reduced due to the efficient heat dissipation by the fluid surrounding the sample. When illuminated with green light of 505 nm the system recovers its original shape confirming the photochemical origin of the deformation.

Beyond contraction under load, photoinduced bending of uniaxially aligned printed LCE strips has been assessed underwater. Significant bending of the uniaxial strips was achieved within a sub second timescale under UV light, with no measurable relaxation immediately after switching off the UV light. The phenomenology observed confirms the predominant photochemical origin of our deformation having its origin in the population gradient across the film thickness of *cis* isomers, that disrupt the LC order and generates stress within the film.

The developed azobenzene containing macromers with low liquid crystal to isotropic transition temperature has enabled the digital generation of complex director textures. We harnessed this capability to craft a biomimetic LCE swimmer that closely resembles a four‐lapped ephyra. When irradiated underwater with UV light from an LED, the four lappets synchronously and rapidly bend toward the light leading to swimmer propulsion away from the light source. The original shape can be recovered by illuminating with green light for a few seconds. A net vertical displacement results from this sequential UV‐green illumination cycle. Iteration of these illumination cycles consistently propels the swimmer away from the light source.

Our system, whose underwater deformation relies on a photochemical contribution, quickly responds to moderate‐power LED light sources without requiring the use of a focused laser, which is typically needed for heating a specific part of LCEs when relying on photothermal actuation. Thanks to this sensitivity, a larger area can be covered with sufficient light intensity using a conventional light source. This enables illumination of the entire sample and its surroundings to drive swimming, eliminating the need for a tracking system to follow its movements and produce propulsion by precisely illuminating a specific point of the actuator. Looking ahead, we anticipate the possibility of groups of LCE swimmers, collectively influenced by the same extended light beam, swimming synchronously with sufficient area coverage or through further miniaturization. Importantly, the predominantly photochemical actuation of our system eliminates the need for heating the LCE to induce bending and swimming motion. This unique characteristic holds potential for future applications in biomedicine and cell culture.

## 4 Experimental Section

### Materials

Bifunctional monomer 4‐(6‐(Acryloyxylhehyloxyl)phenyl 4‐(6‐(acryloyloxy)hexyloxy)benzoate (C6BAPE), and bifunctional azobenzene 4,4′‐Bis(9‐(acryloyloxy)nonyloxy)azobenzene (A9A) were purchased from SYNTHON Chemicals GmbH & Co. KG. Chain extender 2,2′‐(ethylenedioxy)diethanethiol, and catalyser triethylamine (TEA) were purchased from Sigma–Aldrich. Photoinitiator phenylbis(2,4,6‐trimethylbenzoyl)phosphine oxide (IRG819) was purchased from Ciba Specialty Chemicals Inc. Solvents acetone and dichloromethane (DCM) were purchased respectively from Chem‐Lab NV and Scharlab,S.L. Hydrochloric acid (HCl) and magnesium sulfate (MgSO_4_) for the washing process were purchased from Fisher Scientific. 

### Synthesis Process

The LCE precursor was formed following a thiol‐acrylate Michel addition of a 1:1.15 thiol to acrylate mixture. We have generated Three different macromers, varying the molar ratios of the diacrylates C6BAPE and A9A, 95:5, 90:10, and 85:15, were generated. The synthesis process was similar to the one proposed by Roach at al.^[^ ^60^ ^]^ and it starts by dissolving the C6BAPE and A9A into acetone. Once dissolved, EDDET and TEA (5 wt.%) are added to the mixture and the solution was kept stirring with hot plate temperature at 70 °C for 3 h and then put in a vacuum chamber at 70 °C overnight. The macromer was dissolved in DCM, washed with an HCl (1 m) and brine. The macromer solution was dried with anhydrous MgSO_4_ and the solvent was evaporated.^[^ ^58^ ^]^ At this point, the macromer was dissolved again in DCM and 5 wt% of IRG819 was added to the mixture. The final ink was obtained after evaporating the remaining solvent (heating under vacuum at 70 °C overnight). 

### 4D Printing and Curing Process of LCE Elements

The printing step of the samples was conducted using a homebuilt 3D printer assembled in the laboratory, as previously described by López‐Valdeolivas et al.^[^ ^47^ ^]^ Essentially, a computer numerical control (CNC) router chassis was coupled with the ink reservoir. This was temperature‐controlled and pressure was applied on demand to extrude the ink through the needle while X, Y, and Z motions were executed. AutoCAD 2023 software was used to generate the design files then converted to G‐code, by a home‐made software, for a correct interface with the printer.

The previously synthetized ink was loaded in a syringe and extruded through a 23‐gauge (330 mm inner diameter) needle tip. The printing head was heated to 75 °C and the microscope slides, with a PVA coating (150 nm thick), used as a substrate were kept at RT on the printer flatbed. To prepare PVA coated substrates, a 5 wt% PVA solution in Milli‐Q purified water was prepared and applied on the glass slides by spin‐coating (1800 r.p.m. for 60 s). The coating was left to dry at 60 °C for 60 min.^[^ ^53^ ^]^ Printing speeds of 10 to 12 mm s^−1^ and a pressure of 7 bar were used, while the relative height of the tip of the needle in relation to the surface was adjusted before every printing session. Typically, a 300 to 500 µm distance from the needle tip to the printing surface was set. 

Two main different geometries were used for the sample preparation: a rectangular strip (17 mm x 3 mm, distance between lines of 300 µm) used to perform the reference bending actuation, and a cross‐shaped sample (17 mm x 17 mm, width of each arm 3 mm, distance in between lines 300 µm) for the biomimetic ephyra‐like swimming system. All the samples were composed of three layers, typically with a thickness of 20 ± 2 µm each, measured using a Bruker Dektak XT Stylus Profiler. 

After the printing step, the samples were cured by exposure to 430 nm light from a LED source (Thorlabs Inc.) with a power of 21 mW cm^−2^. This photocuring was carried out under mild vacuum (100 mbar) for 15 min for each side of the sample at the end of the printing process. For multilayer samples, light exposure was performed for 10 min each printed layer, and additionally 15 min for each side of the sample at the end of the printing process of the last layer. To perform gel fraction tests, the cured sample was initially weighted, then submerged for 24 h in THF, dried, and then finally weighted again. The gel fraction was calculated as a percentage by dividing the final weight of the insoluble gel by the initial weight of the sample and then multiplying by 100. 

### Mechanical Testing

The elastic module of uniaxially printed and cured rectangular samples (40 ± 2 µm of thickness) was tested, before UV irradiation, and after UV irradiation. A force transducer was used to measure the force during the sample stretching by controlled movement of a motorized stage holding the sample.

### Polarization Optical Microscope (POM)

The structures were examined using a Nikon Eclipse 80I POM.

### Spectroscopic Characterization

Absorption spectra were measured using a UV–vis–NIR Cary500 spectrophotometer. Measurements were carried out on a printed monoaxial layer (20 µm) sample, three times: after the curing step, after 10 s of irradiation under 365 nm light (100 mW cm^−2^), and finally after 10 s of irradiation under 505 nm light (100 mW cm^−2^). 

### Differential Scanning Calorimetry (DSC)

Thermal transitions were determined by DSC using a TA DSC Q‐2000 instrument under a nitrogen atmosphere with powdered samples (≈3 mg) sealed in aluminum pans. Glass transition temperatures were determined at the midpoint of the baseline jump, and the mesophase‐isotropic phase transition temperature was read at the maximum of the corresponding peaks.

### Nuclear Magnetic Resonance

NMR spectra were recorder on Bruker Avance spectrometers operating at 400 / 500 MHz for ^1^H and 100 / 125 MHz for ^13^C using standard pulse sequences. Chemical shifts are given in ppm.

### FTIR

Spectra of the ink and the LCE were recorded on a Bruker Vertex 70 FT‐IR spectrophotometer equipped with an Specac Golden gate diamond ATR, and 50 scans at a resolution of 4 cm^−1^ were employed.

### Thermoactuation Characterization

Temperature induced contraction tests were performed on uniaxially oriented LCE printed samples (17 mm x 3 mm x 60 µm, 3 layers) fixed to a sample holder in one extreme. A weight of 1 g was attached by a Kapton tape to the other extreme of the sample, that was then vertically suspended. Holder, sample, and weight are immersed in a metallic chamber filled with water. The chamber had two opposing glass slides facing each other to allow sample observation. A thermocouple was immersed in the fluid to monitor the temperature of the water. The whole system was successively heated by using a temperature‐controlled heating plate. Pictures of the sample were taken every 2.5 °C to characterize the actuation were taken using a digital camera Nikon D5200, and the samples dimensions changes analysed by ImageJ software. These tests were repeated for cured samples of the three different LCEs with 5, 10, and 15 mol% of A9A. 

### Photoactuation Characterization

The experimental setup for the photoinduced contraction tests was the same as the one described on the previous section on thermoactuation characterization. The sample, immersed in water, was first irradiated with 365 nm light (100 mW cm^−2^) for 2 min and 30 s, and successively with 505 nm light (100 mW cm^−2^) again for 2 min. The sample was filmed throughout the entire irradiation experiment using the same digital camera mentioned above, and the video was then analyzed using ImageJ software to quantify the photoinduced contraction. As in the thermoactuation characterization experiments, these tests were repeated for the three different LCEs with 5, 10, and 15 mol.% of azobenzene. 

For the photoinduced bending tests, a uniaxially oriented LCE printed strips (17 mm x 3 mm) of 1, 2, 3, and 4 layers (≈20, 40, 60, and 80 µm, thick, respectively) with 15 mol% of azobenzene were explored. The sample was clamped and suspended in a nearly horizontal orientation underwater. Subsequently, it was irradiated with 365 nm (100 mW cm^−2^) and 505 nm (40 mW cm^−2^) light, both illuminating the sample from the bottom. In the first test, which aimed to assess maximum bending ability and reversibility, the sample was exposed to UV light until it reached equilibrium. Following this, it was exposed to 505 nm light until its complete initial shape was restored. For the second test, which focused on assessing the reproducibility and repeatability of the bending‐unbending process just the three‐layer sample was used, the 505 nm light was kept switched on throughout all the experiment. Meanwhile, the UV light was switched on for 1 s and off for 5 s, making a total of 20 cycles. After the final cycle, the sample was exposed to 505 nm light irradiation for 10 s to facilitate complete sample recovery. Pictures and videos were subsequently analyzed to determine the bending angle and the reversibility of the actuation across the different irradiation cycles.

### Swimming Test

These tests were performed using the printed biomimetic ephyra‐like LCE system described above (60 µm of thickness, three layers) with 15 mol.% of azobenzene. The sample was immersed in a tank (25 × 25 × 25 cm) filled with water. The density of water was adjusted to 1.2 g cm^−3^, slightly below that of the LCE sample, with the addition of sugar. The four‐lappet ephyra‐like LCE sample, positioned horizontally at the bottom of the tank, was then irradiated from the bottom with alternating collinear 365 nm (100 mW cm^−2^ for 1.5 s) and 505 nm (40 mW cm^−2^ for 3.5 s) light beams with a diameter of 5 cm. The employed solution for the swimming tests presents an absorption of 0.027 in a 1 cm cuvette at 365 nm, the excitation wavelength for lappet bending (see Figure S10,Supporting Information). This means that 6% of the UV light reaching the cuvette was absorbed by the solution in its path through the cuvette. Given the swimmer's displacement of ≈2 cm in the fluid tank experiments (see figure 4E), the light reaching the sample remains nearly constant, varying within 12% during the swimming experiments. While the attenuation was minimal in the conducted experiments, it should be considered if the light path to the sample becomes larger. This attenuation could result in variations in the bending dynamics of the lappets as the swimmer moves farther away from the light source.

The change in height *h* of the swimmer, using the bell apex as reference, was tracked in the video as a function of time *t* by capturing frames at intervals of time Δ*t* of 250 ms. From the height measurements at different moments, the vertical velocity *v* was calculated as:

(2) $$v\;\left(t\right)=\;\frac{h\left(t+\Delta t\right)-\;h\left(t\right)}{\Delta t}$$

Similarly, taking the speed calculated as a function of time, acceleration *a* was calculated as:

(3) $$a\;\left(t\right)=\;\frac{v\left(t+\Delta t\right)-\;v\left(t\right)}{\Delta t}$$

### Medium Viscosity Characterization

The viscosity of the swimming medium was measured using an Ostwald U‐ shaped capillary viscometer, with distilled water serving as the reference liquid.

## Conflict of Interest

The authors declare no conflict of interest.

## Supporting information

Supporting Information

Supplementary Movie 1

Supplemental Movie 2

Supplemental Movie 3

## Data Availability

The data that supports the findings of this study are openly available in Zenodo at https://zenodo.org/communities/storm‐bots‐itn
