# Supplementary material for: Photochemically Induced Propulsion of a 4D Printed Liquid Crystal Elastomer Biomimetic Swimmer
Source: Adv Sci (Weinh). 2024 Apr 8;11(25):2308561. doi: 10.1002/advs.202308561 (PMC11220691; doi:10.1002/advs.202308561)
Supplement: Supplementary file 1 — Supporting Information [file ADVS-11-2308561-s003.pdf]

ADVANCED SCIENCE
Open Access

# Supporting Information

for *Adv. Sci.*, DOI 10.1002/advs.202308561

Photochemically Induced Propulsion of a 4D Printed Liquid Crystal Elastomer Biomimetic Swimmer

*Paolo Sartori, Rahul Singh Yadav, Jesús del Barrio, Antonio DeSimone and Carlos Sánchez-Somolinos**

# Supporting Information

## Photochemically Induced Propulsion of a Four-Dimensional Printed Liquid Crystal Elastomer Biomimetic Swimmer

*Paolo Sartori, Rahul Singh Yadav, Jesús del Barrio, Antonio De Simone, and Carlos Sánchez-Somolinos**

## Supporting Figures

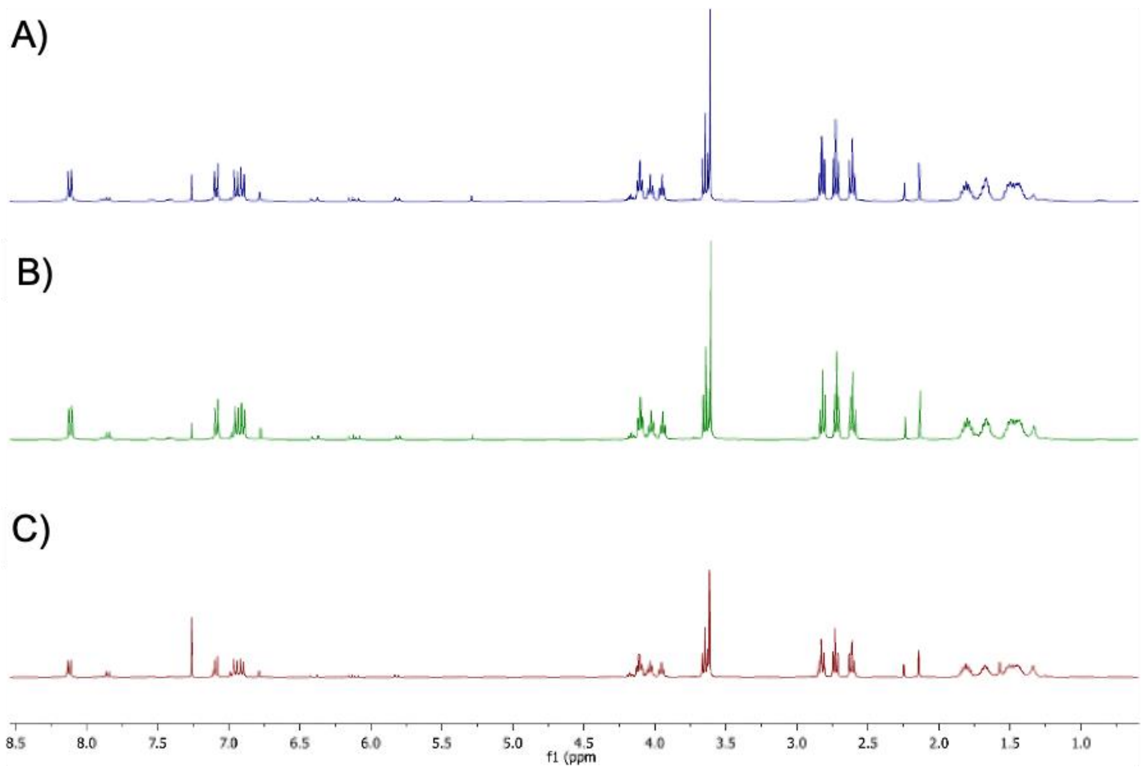


**Figure S1:** $^{1}$H NMR spectra of the liquid crystal inks: 5 (A), 10 (B) and 15 mol% (C) of A9A.

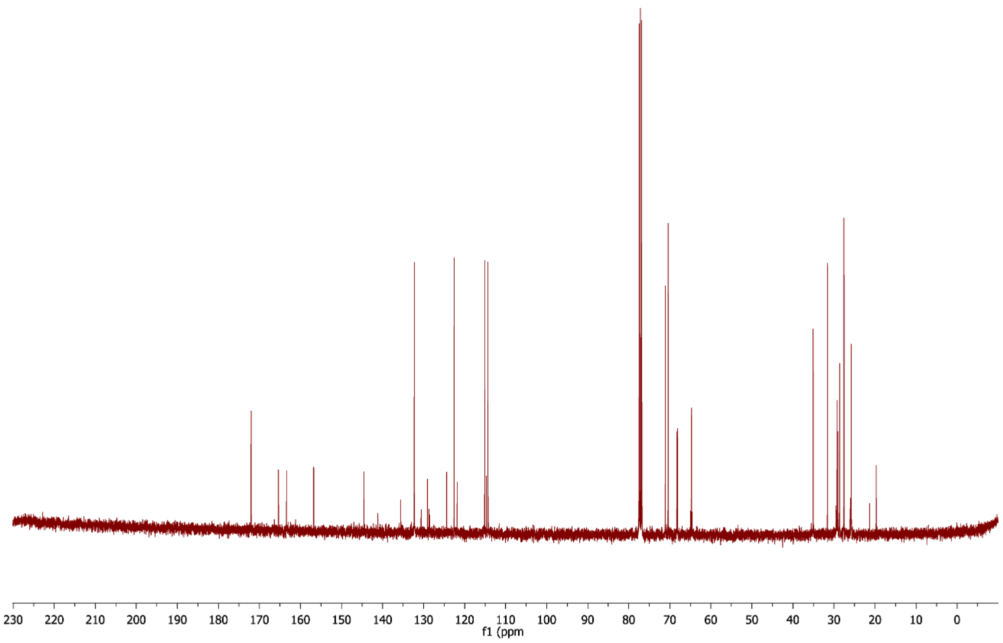


**Figure S2**: $^{13}$CNMR spectrum of the 15 mol% of A9A.

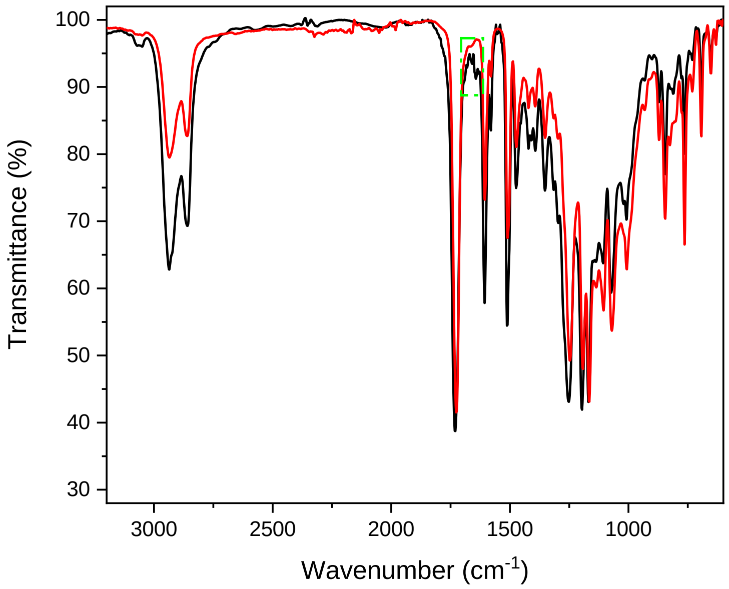


**Figure S3:** FTIR of 15 mol% A9A ink (black line) and 15 mol% A9A elastomer (red line). The green dashed line box corresponds to the band at ca. 1635 – 1640 cm-1, characteristic of the -C=C- bond of the acrylate groups.

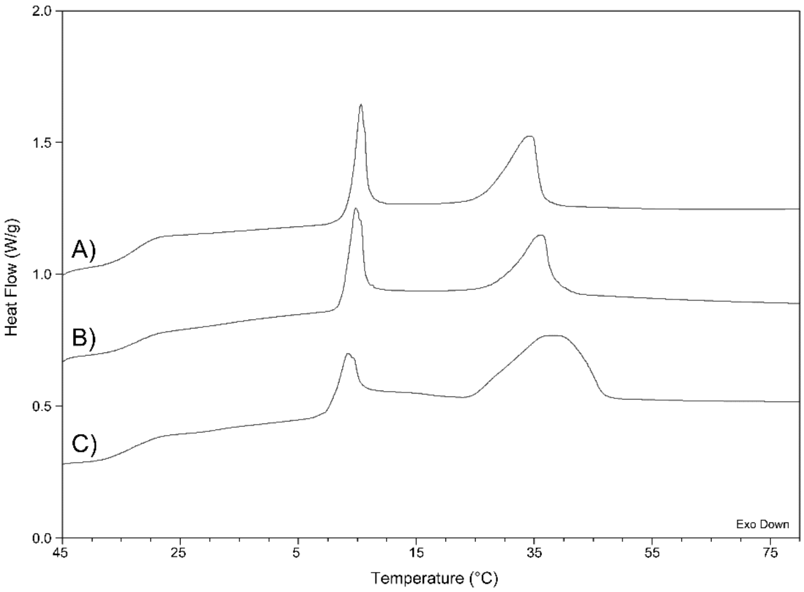


**Figure S4**: DSC traces of the uncured inks with 5 (A), 10 (B) and 15 mol% (C) of A9A. The high temperature endothermic peak in each of the DSC traces is associated with the liquid crystal to isotropic transition.

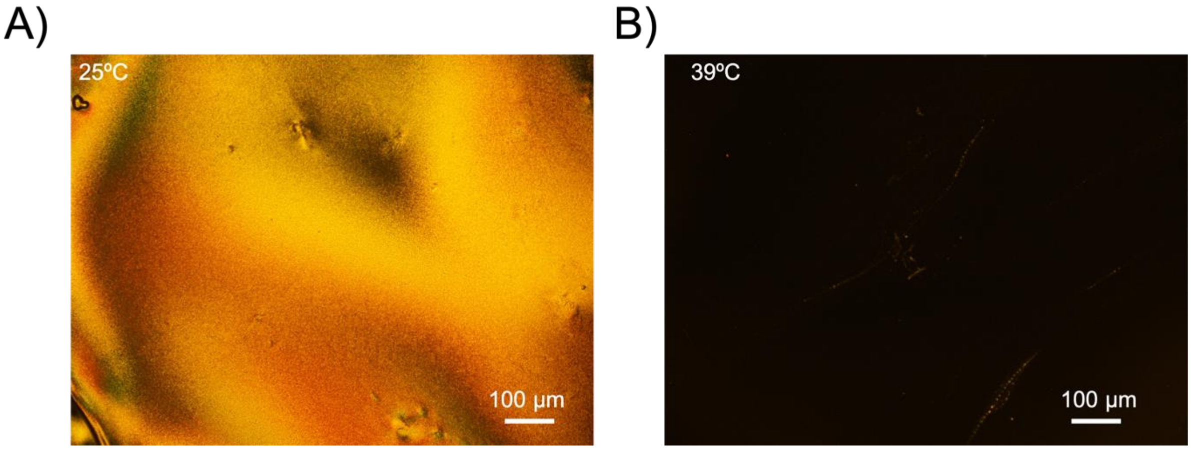

**Figure S5**: POM images of the 15 mol% A9A ink at 25 ºC (A) and at 39 ºC (B). Scale bars: 100 μm.

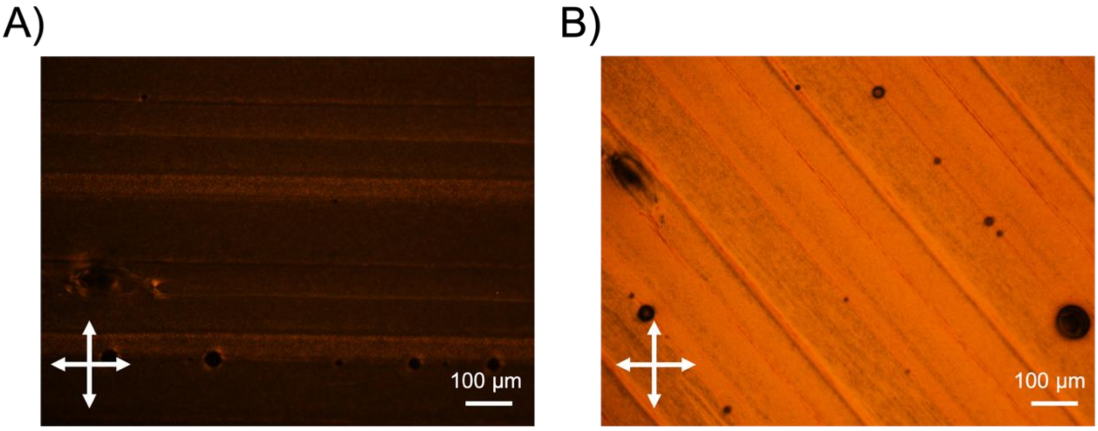


**Figure S6**: POM images of closely printed lines, forming a continuous uniaxially oriented layer, after photopolymerization (15 mol% A9A LCE). Images are acquired with the sample between crossed polarizers. White crosses indicate polarizer transmission directions. The printing direction is oriented A) parallel and B) at 45° with respect to the first polarizer transmission direction. Scale bars: 100 μm.

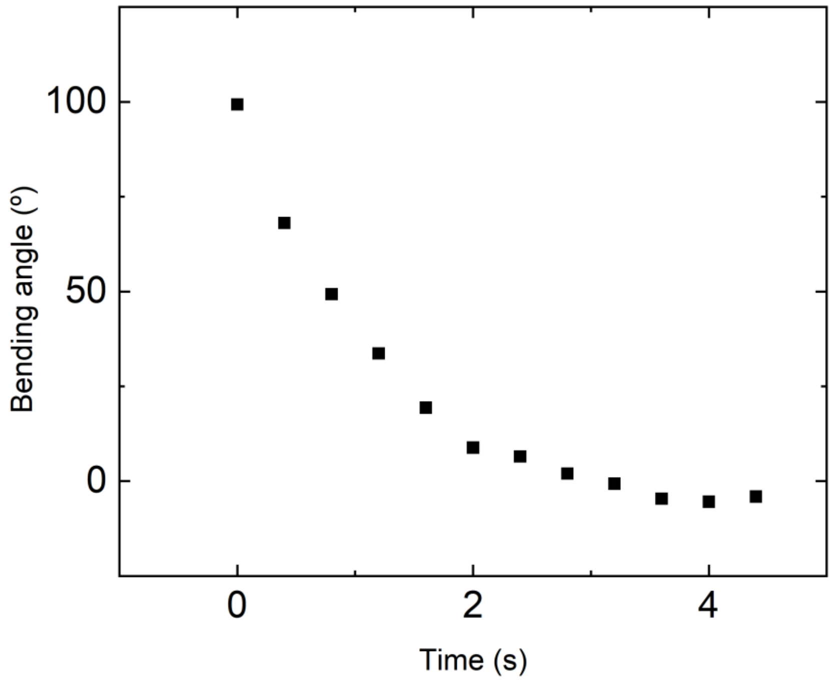


**Figure S7:** Angle of the tip as a function of time of a 4-layer strip (80 μm thick) under constant UV irradiation (100 mW·cm$^{-2}$). Angle is defined as in Figure 3B of the main manuscript.

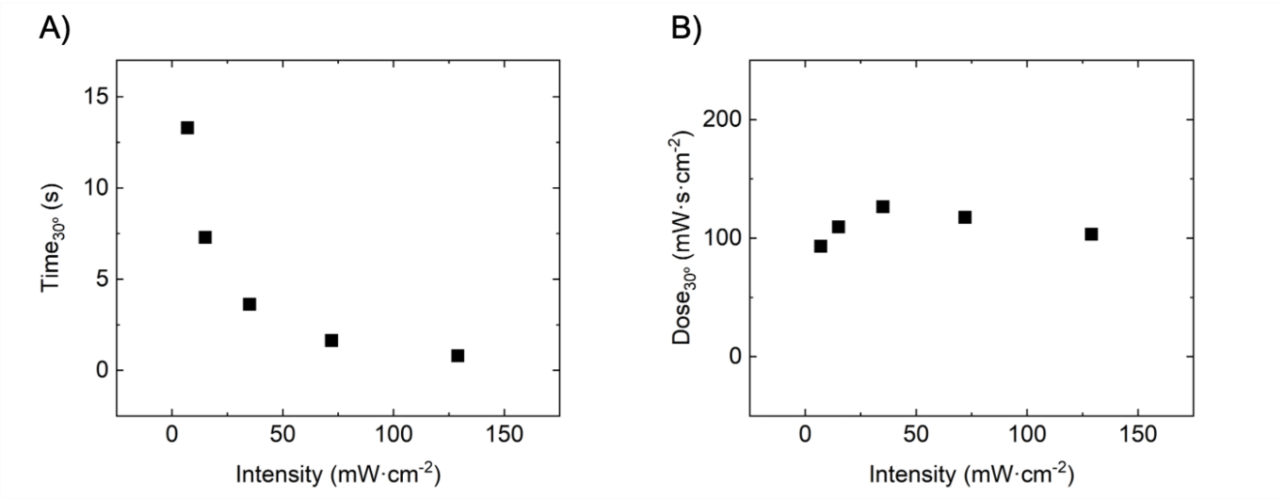


**Figure S8:** (A) Time needed for the tip to reach 30º from the vertical direction for different intensities of light; and (B) needed dose (intensity times irradiation time) as a function of the applied intensity.

**Table S1:** Evaluation of radius of radius of curvature 1/$r$ on the basis of estimated photodeformation $\varepsilon_p = \frac{L - L_0}{L_0}$, using Timoshenko's Eq.(1) in the main text (see Figure S9 below).

| $\varepsilon_p$ | $m = \frac{d}{t}$ | $r$ [$mm$] |
|---|---|---|
| **0.0005** | 10/60 | 16.3 |
| 0.01 | 10/60 | 8.2 |
| 0.016 (1s) | 10/60 | 5.1 |
| 0.025 (2s) | 10/60 | 3.3 |

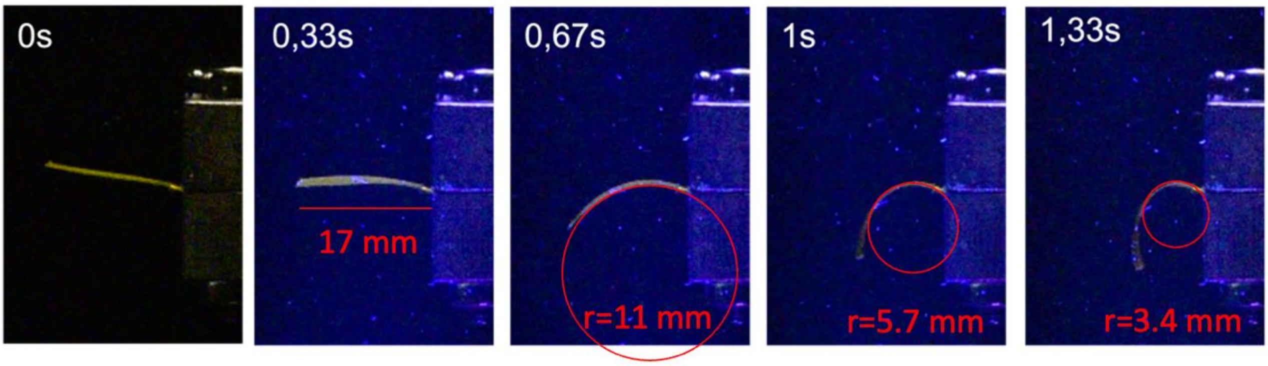


**Figure S9:** Estimation of curvature 1/$r$ in experiments obtained from the radius $r$ of the osculating circle to the mid-axis of the sample near the clamp. Pictures included are those of Figure 3 of the main text and correspond to a 3-layer strip (60 μm thick) of 15 mol% A9A LCE with uniaxially oriented director along the long side of the actuator.

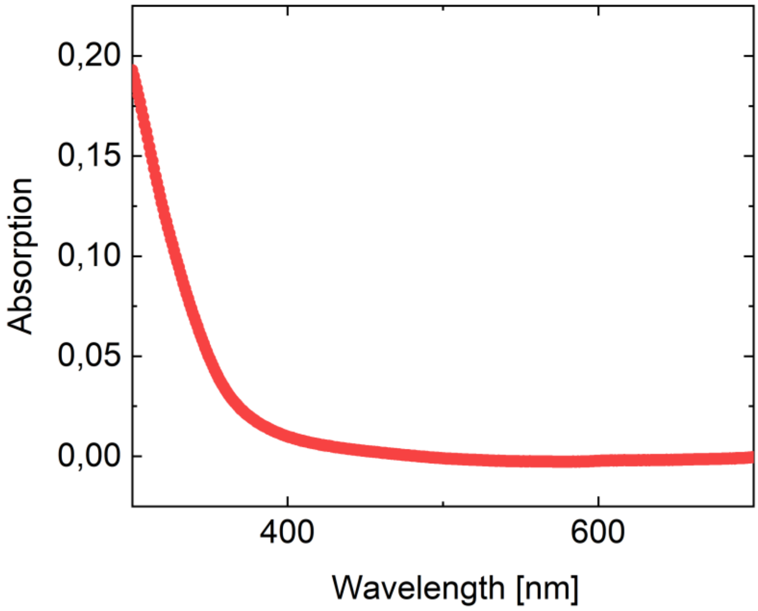


**Figure S10**: UV-Vis absorption spectrum of the water and sugar solution (1 cm path length cuvette).

**Supporting Movies**

**Movie S1**: Fast bending and recovery of a 3 layered (60 µm thick) horizontal LCE strip (15 mol% A9A) under 365 nm (100 mW cm$^{-2}$) until the equilibrium is reached, and 505 nm light irradiation (40 mW cm$^{-2}$) until the complete recovery of the sample's shape.

**Movie S2**: Cyclic bending and recovery of a 3 layered (60 µm thick) horizontal LCE strip, under constant 505 nm (40 mW cm$^{-2}$) light and alternated 365 nm (100 mW cm$^{-2}$) light (1 s ON and 5 s OFF). After 20 cycles, the sample is left under 505 nm light until complete initial shape recovery.

**Movie S3**: Free swimming of a biomimetic ephyra-like LCE sample, (3 layers, 60 µm thick, 15 mol% A9A) under alternated 365 (100 mW cm$^{-2}$, 1.5 s) and 505 nm (40 mW cm$^{-2}$, 3.5 s) light exposure.
